# UAV-Based Coverage Path Planning for Unmanned Agricultural Vehicles

**DOI:** 10.3390/s26030927

**Published:** 2026-02-01

**Authors:** Guangjie Xue, Engen Zhang, Guangshun An, Juan Du, Xiang Yin, Peng Zhou, Xuening Zhang

**Affiliations:** 1 School of Agricultural Engineering and Food Science, Shandong University of Technology, Zibo 255000, China; 18554516779@163.com (G.X.); zeg_nice2mu@163.com (E.Z.); azl17613@163.com (G.A.); dujuan@sdut.edu.cn (J.D.); zhoupeng@sdut.edu.cn (P.Z.); zxn@sdut.edu.cn (X.Z.); 2 Shandong Provincial Key Laboratory of Smart Agricultural Technology and Intelligent Agricultural Machinery Equipment for Field Crops, Zibo 255000, China

**Keywords:** agricultural vehicles, UAV, coordinate transformation, path planning

## Abstract

Accurate path planning was the prerequisite for autonomous navigation of agricultural vehicles. An Unmanned Aerial Vehicle (UAV)-based coverage path planning was developed in this research for automating guidance of agricultural vehicles and reducing the operator maneuver in the creation of navigation maps. High-resolution orthophoto maps of the field were constructed by using low-altitude UAV photogrammetry to obtain spatial information. Travel paths and working paths were automatically generated from anchor points selected by the operator under the image coordinate domain. The navigation path for unmanned agricultural vehicles was generated by Mercator projection-based conversion for the anchor pixel coordinates into latitude and longitude geographic coordinates. A Graphical User Interface (GUI) was developed for path generation, visualization, and performance evaluation, through which the proposed path planning method was implemented for autonomous agricultural vehicle navigation. Calculation accuracy tests demonstrated the mean planar coordinate error was 2.23 cm and the maximum error was 3.37 cm for path planning. Field tests showed that lateral navigation errors remained within ±5.5 cm for the unmanned high-clearance sprayer, which indicated that the developed UAV-based coverage path planning method was feasible and featured high accuracy. It provided an effective solution for achieving fully autonomous agricultural vehicle operations.

## 1. Introduction

With the advancement of modern scientific and technological fields—including sensor and measurement-control technologies, communication technologies, intelligent control systems, as well as the application of machine vision and Global Navigation Satellite System (GNSS) navigation used in autonomous driving—modern agricultural production has been rapidly progressing toward greater intelligence, informatization, large-scale operation, and precision [1,2,3]. Autonomous navigation technology in agricultural vehicles was recognized as a core component within the precision agriculture technology system, and it has played a critical role in the realization of unmanned farming operations [4,5,6].

Although intelligent agricultural machinery technology is advancing rapidly, path planning techniques for autonomous navigation of agricultural vehicles remain an aspect that needs to improve, especially when confronted with the complex and highly variable conditions of real-world farming environments. Extensive research has been conducted on coverage path planning and navigation for agricultural vehicles [7,8,9,10]. An improved ant colony algorithm was employed by Tu et al. to reduce path planning costs, which provided important references for addressing collaborative scheduling problems among agricultural machinery during field operations [11]. Numerous scholars have also conducted research on path tracking and control. To address the sideslip problem of rice farm machinery in paddy field environments, a model predictive control (MPC) path-tracking method based on attitude correction for agricultural machinery was proposed, and field experiments demonstrated that the average root mean square error for three-line straight-path tracking was 0.043 m [12]. A path-tracking algorithm for agricultural machinery based on optimal target points was proposed, which simulated the driver’s look-ahead behavior to identify the optimal target point within the look-ahead region according to an evaluation function, and tracking error was reduced by over 20% compared to the pure pursuit algorithm [13]. A dynamic turning path planning method for four-wheel vehicles based on an asymmetric switching steering strategy was proposed. When agricultural vehicles experienced field slippage, this algorithm could dynamically replant the path according to the real-time position of the vehicle [14]. To address the issues of low trajectory planning efficiency and susceptibility to local optima for unmanned agricultural machinery in complex, narrow, and unstructured environments, this research proposed an improved bidirectional A* algorithm combined with an optimal control method [15]. The study proposed a single-obstacle avoidance algorithm based on agricultural machinery motion rules, as well as dual/multiple obstacle avoidance algorithms determined by the dimensions of the safe driving area [16].

Numerous scholars have investigated classical algorithms, theoretical foundations, coverage path planning, navigation, as well as path tracking and control for agricultural vehicles. These research efforts have been complemented by the growing application of UAVs in modern agricultural production, which is accelerating the development of unmanned farms [17,18,19,20]. This research analyzed the latest advances in UAV-ground vehicle collaboration, including collaborative navigation, perception fusion, and task allocation in agricultural applications [21]. A UAV-ground vehicle collaborative precision spraying system for honey pomelo orchards was developed in this research, where UAVs were responsible for canopy-top spraying while ground vehicles handled trunk and lower-canopy application, thereby improving pesticide utilization efficiency [22]. Researchers proposed an air-ground collaborative 3D mapping framework in this paper, where UAVs provide aerial perspectives and ground vehicles capture close-range details, generating a high-precision 3D model of the fields [23]. This research explored the collaborative applications of UAVs and ground vehicles within vineyard environments, encompassing agricultural operations such as monitoring, pruning, and harvesting. A decentralized multi-phase approach was proposed as an alternative to more common cooperative schemes. When perennial crops are considered, it is advantageous to build a simplified geometrical crop model. Preliminary results highlight the benefits achievable by exploiting the tailored technologies selected and applied to improve each of the analyzed mission phases [24].

Existing path planning methods for agricultural vehicles often require extensive manual intervention, which limits the applicability to fully autonomous operation. To address the above issues, this research proposed a UAV-based coverage path planning method for unmanned agricultural vehicles. High-resolution orthophoto maps obtained from low-altitude UAV photogrammetry were used to generate a travel path and working path, while a Mercator projection-based coordinate transformation was designed to convert pixel-based anchor points into geographic coordinates for the navigation of unmanned agricultural vehicles. Field tests with an unmanned high-clearance sprayer were conducted to validate the feasibility of the proposed method.

## 2. Materials and Methods

**Figure 1:**
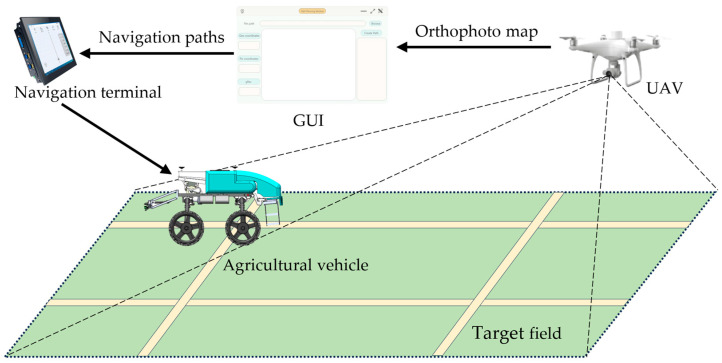
UAV-based path planning for full coverage of the target field.

As illustrated in Figure 1, a photogrammetric UAV was used to obtain aerial images of the target field, which were stitched to generate a high-resolution orthophoto map. Path anchor points were calibrated to plan travel paths and working paths according to the generated orthophoto map. A coordinate transformation algorithm was developed to convert anchor pixel coordinates into geographic coordinates for the generation of navigation paths for unmanned agricultural vehicles.

**Figure 2:**
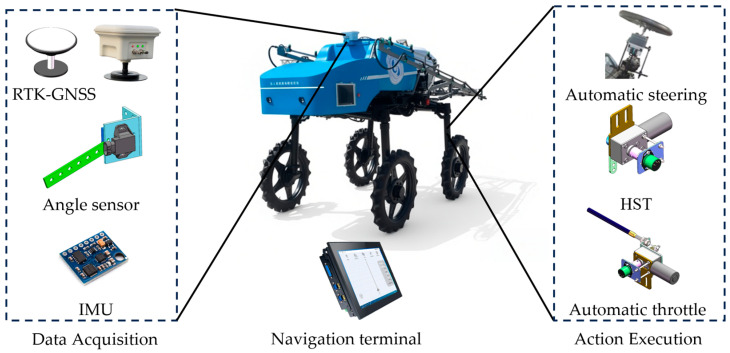
Main components of the unmanned high-clearance sprayer.

A high-clearance sprayer with the autonomous navigation system [25] was used as the test platform, as shown in Figure 2. Its main parameters were shown in Table 1. The system contains data acquisition, action execution, and a navigation system. Data acquisition was realized using a positioning module, angle sensor, and Inertial Measurement Unit (IMU), while motion execution was achieved through automatic steering, an automatic throttle, and a continuously variable transmission. These modules are coordinated by the navigation control system to enable unmanned operation. The navigation system employed a dual-antenna positioning and orientation receiver based on a Trimble BD982 board with Real-Time Kinematic (RTK) differential service, together with an IMU.

A Phantom 4 RTK UAV was used as the low-altitude photogrammetric platform for aerial imaging of the operational areas. The UAV supported control-point-free aerial surveying and provided centimeter-level positioning accuracy while maintaining high-resolution imaging performance. It was equipped with an RTK positioning module and a Time Sync system, which enabled microsecond-level synchronization among the flight controller, camera, and RTK module, thereby reducing temporal errors between image acquisition and positioning data. The key performance parameters of the UAV are shown in Table 2.

### 2.1. Acquisition of Field Orthophoto Map

To enable accurate planning of travel paths and working paths for unmanned agricultural vehicles, low-altitude aerial photogrammetry was conducted using a Phantom 4 RTK UAV (SZ DJI Technology Co., Ltd. in Shenzhen, China) to generate a high-precision orthophoto map of the target field. A set of overlapping aerial images was acquired and subsequently processed through photogrammetric reconstruction to produce a geometrically corrected orthophoto map with unified scale and spatial reference, which served as the fundamental spatial data source for subsequent path planning and coordinate transformation. The field orthophoto map was constructed through the following procedures:

(1) Definition of the target field and takeoff–landing site selection:

The target field was firstly defined to include the garage. Within the target field, a location with an unobstructed view and minimal electromagnetic interference was selected as the UAV takeoff and landing site. In addition, an arbitrary point within the target field was designated as a reference point, and its geographic coordinates (latitude and longitude) were recorded for use in subsequent coordinate transformation and accuracy evaluation.

**Figure 3:**
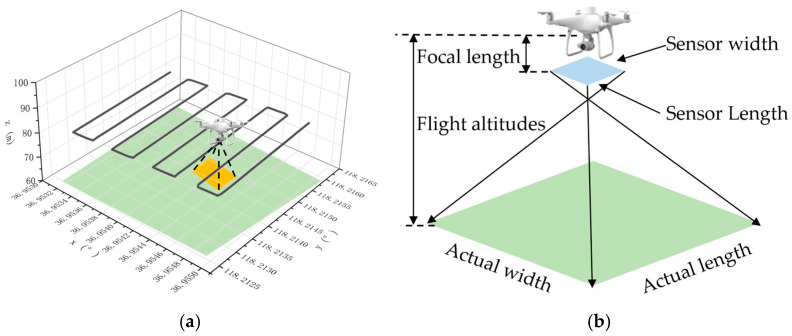
UAV flight paths and sensor–ground mapping geometry: (**a**) UAV flight paths over the target field and (**b**) relationship between sensor parameters and ground distance.

(2) UAV flight planning and image acquisition:

The UAV flight paths were planned in accordance with the Low-Altitude Digital Aerial Photogrammetry Fieldwork Specifications (CH/T 3005-2021) [26]. Appropriate flight altitude, forward overlap, and side overlap ratios were configured to ensure sufficient image redundancy. The UAV then operated autonomous flight missions to acquire high-resolution aerial images covering the entire area. The UAV flight paths and the relationship between the camera sensor parameters and ground distance are shown in Figure 3.

(3) Image processing and orthophoto map generation:

The corresponding latitude, longitude, and altitude information were extracted from the Exchangeable Image File Format (EXIF) metadata for each aerial image. Feature points were then detected and matched between adjacent images to generate point cloud data for the entire surveyed area. By integrating the Position and Orientation System (POS) data with the feature matching results, the exterior orientation parameters of each image and the three-dimensional coordinates of ground points were calculated through bundle block adjustment, thereby establishing an accurate geometric relationship between the images and the ground. Based on the reconstructed point cloud, a Digital Surface Model (DSM) of the field was generated, followed by orthorectification and image mosaicking to produce the high-resolution orthophoto map of the target field. The mosaicking process involved automatic feature extraction and matching across overlapping images, followed by bundle adjustment constrained by the onboard Real-Time Kinematic (RTK) positioning data. Geometric correction was achieved through orthorectification using the generated DSM. The accuracy of the resulting orthophoto was indirectly validated by the coordinate transformation accuracy tests.

### 2.2. Planing of Travel Paths and Working Paths

Travel paths and working paths for the agricultural vehicle were planned according to the pixel coordinate system of the orthophoto map. Travel paths included forward paths from the garage to the starting position of the working field and return paths from the field exit back to the garage. According to the operational characteristics of the high-clearance sprayer used in this research, the working paths were generated as rectangular strip patterns by delineating the field plots.

For the planning of forward and return paths, the locations of the garage, the starting point, and the ending point of the working field were first determined. Path anchor points were then calibrated along the roads connecting the garage to the field entrance and from the field exit back to the garage on the orthophoto map. The spacing between adjacent anchor points was selected according to practical requirements. Smaller spacing resulted in a larger number of anchor points, which improved tracking performance of the agricultural vehicle, whereas larger spacing reduced data volume and facilitated subsequent processing. The spacing was determined based on road width, with narrower roads requiring denser anchor points.

For the generation of working paths, the target field was identified by selecting its boundary points on the orthophoto map to obtain pixel coordinate information. Based on the geometric dimensions of the field, the vehicle working width, minimum turning radius, and operation direction were used as input parameters to generate coverage paths.

Using the high-clearance sprayer as the test platform, an automatic path generation algorithm was developed for working path planning. The sprayer had a minimum turning radius of 3.5 m and a working width of 12 m. By default, the primary working direction was aligned with the longer side of the working field to minimize the number of turns. After determining the primary working direction and the lateral pixel dimension of the field, the required number of working swaths was calculated based on the relationship among the field width, working width, and minimum turning radius. Given the significant difference between the working width and the minimum turning radius, the number of working swaths was determined using the following formula:

(1) $$Sw=\left\lceil \frac{cAixs\times gRes}{W}\right\rceil$$

where Sw represents the number of working swaths required to ensure complete coverage; cAixs corresponded to the lateral pixel dimension of the working field; gRes signified the ground resolution; W denoted the working width. The ceiling function was applied to ensure no coverage omissions during the spray work of the high-clearance sprayer.

Consequently, the corresponding working swath spacing in the pixel coordinate system was determined, ensuring that working swaths were evenly distributed along the lateral direction of the working field, thereby achieving uniform coverage of the working area. The formula is expressed as follows:

(2) $$aW=\frac{cAixs}{Sw}$$

where aW represented the swath spacing. This formula evenly distributed the lateral pixel dimension, ensuring equal pixel spacing between each working swath. The corresponding actual physical spacing was aW×gRes. This could ensure that the actual swath spacing neither exceeded the working width nor violated the minimum turning radius requirement.

The starting and ending points for each working swath were calculated according to the determined operation starting point and swath spacing. Assuming the coordinates of the operation starting point were (*x*_min_, *y*_min_), the starting and ending point’s coordinate for each working swath were derived as follows:

(3) $$\left\{\begin{array}{l} S_{i}=\left(x_{\min}+i\times aW,y_{\min}\right)\\ E_{i}=\left(x_{\min}+i\times aW,y_{\max}\right) \end{array}\right.$$

where *y*_max_ represented the maximum pixel coordinate along the longer side of the working field; *S_i_* included the starting point coordinates of the corresponding working swath; *E_i_* included the ending point coordinates of the corresponding working swath.

**Figure 4:**
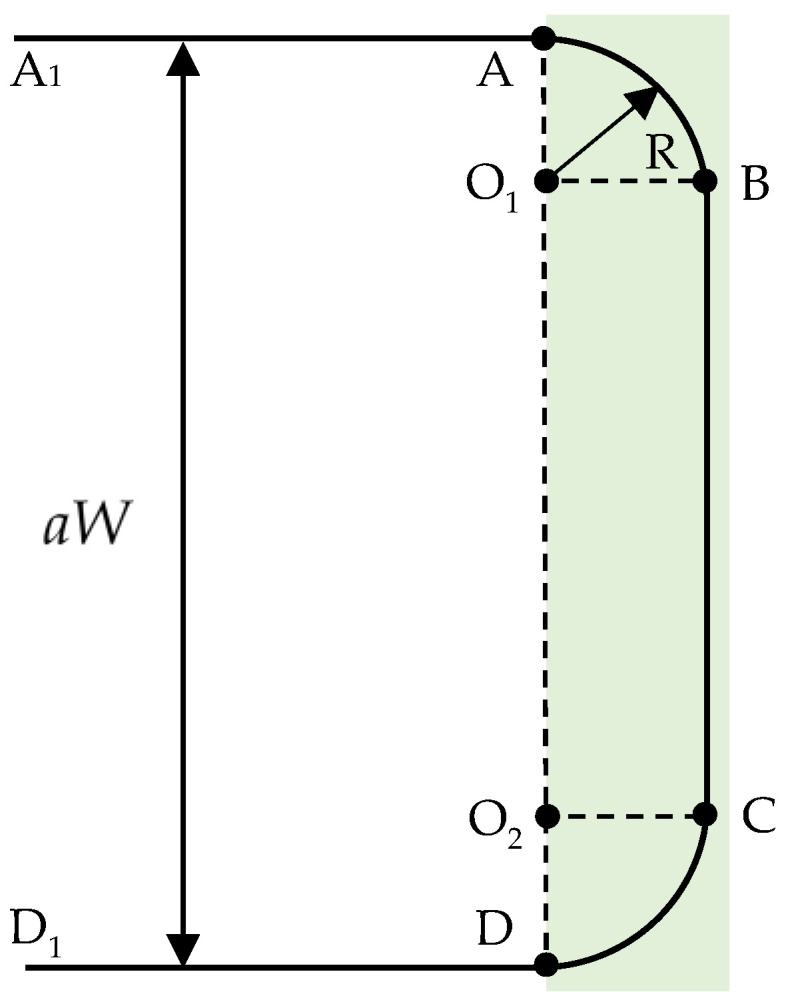
Headland turning of the unmanned high-clearance sprayer.

The headland turning model of the high-clearance sprayer was shown in Figure 4. When the high-clearance sprayer reached the headland after traveling along a working swath, a rectangular turning pattern was executed to enter the next working swath. All coordinate calculations during this process were performed within the pixel coordinate system.

When the high-clearance sprayer reached point *A*, the pixel coordinate of point *B* could be calculated based on the pixel coordinate of point *A*, as shown in Formula (4). The following formula represents the process of coordinate calculation of the point *B*.

(4) $$\left\{\begin{array}{l} L_{AB}=\frac{sqrt(2)\times R}{\mathrm{gR}es}\\ Psi_{AB}=Psi_{A_{1}A}+\frac{\pi }{4}\\ x_{B}=x_{A}+L_{AB}\times \sin Psi_{AB}\\ y_{B}=y_{A}+L_{AB}\times \cos Psi_{AB} \end{array}\right.$$

where $L_{AB}$ represents the length of segment AB; R denotes the turning radius of the high-clearance sprayer; $Psi_{A_{1}A}$ is the angle between the line $A_{1}A$ and the *y*-axis of the pixel coordinate system; $Psi_{AB}$ is the angle between the line AB and the *y*-axis of the image plane coordinate system; $\left(x_{A},y_{A}\right)$ and $\left(x_{B},y_{B}\right)$ are the pixel coordinates of the point A and the point B.

The pixel distance from point *B* to point *C* is (aW−2R), and thus, the pixel coordinate of point *C* can be expressed as follows:

(5) $$\left\{\begin{array}{l} x_{C}=x_{B}\\ y_{C}=y_{B}+\left(aW-\frac{2R}{gRes}\right) \end{array}\right.$$

where $\left(x_{C},y_{C}\right)$ is the pixel coordinate of the point C.

The process of calculating the pixel coordinates of point *D* from the pixel coordinates of point *C* was derived as follows:

(6) $$\left\{\begin{array}{l} L_{CD}=\frac{sqrt(2)\times R}{gRes}\\ Psi_{CD}=Psi_{BC}-\frac{\pi }{4}\\ x_{D}=x_{C}+L_{CD}\times \sin Psi_{CD}\\ y_{D}=y_{C}+L_{CD}\times \cos Psi_{CD} \end{array}\right.$$

where $L_{CD}$ represents the length of segment CD, $Psi_{BC}$ is the angle between the line BC and the *y*-axis of the image plane coordinate system, $Psi_{CD}$ is the angle between the line CD and the *y*-axis of the image plane coordinate system, and $\left(x_{C},y_{C}\right)$ and $\left(x_{D},y_{D}\right)$ are the pixel coordinates of point *C* and point *D*.

Through the aforementioned procedures, the pixel coordinate sets of anchor points for the forward travel path, the working paths, and the return travel path were obtained. These three sets of pixel coordinates were sequentially stored in three separate files.

### 2.3. Generation of Navigation Paths for Agricultural Vehicles

The operational process of agricultural vehicles from initiation to completion could be summarized in three phases. First, the vehicle traveled from the garage to the operation starting point. Subsequently, the vehicle performed the work based on provided parameters such as working width, number of passes, operation direction, operating speed, and headland turning patterns, proceeding until the operation termination point was reached. Finally, the vehicle returned from the operation termination point back to the garage.

The anchor points information of the forward travel paths, working paths, and return travel paths created on the Orthophoto map were stored in three separate files, all in the form of pixel coordinates. Since the navigation system of the vehicle could not directly utilize pixel coordinates, it was necessary to convert the pixel coordinates along the travel paths and working paths into corresponding geographic coordinates (latitude and longitude).

**Figure 5:**
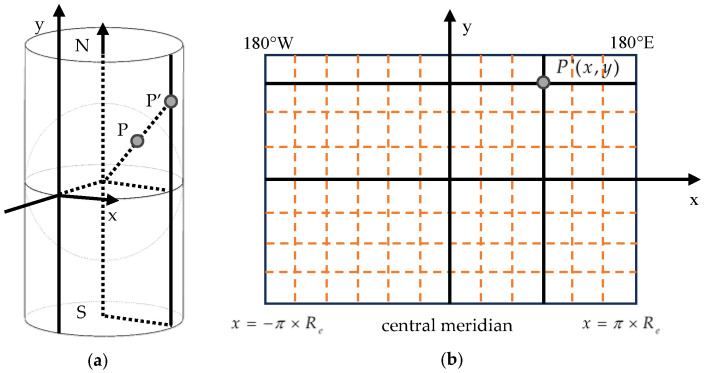
Mercator projection-based conversion from (**a**) the cylindrical reference into (**b**) the plane coordinate system.

The conversion algorithm was designed based on the Mercator projection under a local mapping assumption, as illustrated in Figure 5. The equator was defined as the standard parallel, and the prime meridian was designated as the central meridian. The intersection of these two lines served as the coordinate origin point, with east and north directions assigned as positive, and west and south directions as negative.

The transformation formulas of the projected x-coordinate and y-coordinate are expressed as follows:

(7) $$\left\{\begin{array}{l} x^{t}=R_{e}\lambda \\ y^{t}=R_{e}\times \int_{0}^{\Psi }\sec\Psi d_{\Psi }=R\times \ln\left|\tan(\frac{\pi }{4}+\frac{\Psi }{2})\right| \end{array}\right.$$

where *λ* denotes the longitude of the target point on the Earth’s surface; *R_e_* represents the equivalent Earth radius used for local projection; *Ψ* is the latitude of the target point.

During the algorithm development process, a structure named Sourse was first constructed to store the horizontal and vertical coordinates (x,y), latitude and longitude (lat,lon) of the target pixel points, and the required ground resolution gRes.

By applying Formula (8), the pixel coordinate differences delX and delY between the target point and the reference point were calculated as follows:

(8) $$\left\{\begin{array}{l} delX=x-kPix.x\\ delY=kPix.y-y \end{array}\right.$$

where kPix.x represents the horizontal pixel coordinate of the reference point, and kPix.y represents the vertical pixel coordinate of the reference point.

Since longitude in the Mercator projection exhibits a linear relationship with the projected coordinate system, the formula for calculating the longitude increment between the target point and the reference point could be directly established, the formula is expressed as follows:

(9) $$delLon=\frac{delX\times gRes}{R_{e}\times \cos(\frac{kPLat\times M_{PI}}{180})}$$

where delLon represents the longitude increment; $R_{e}$ denotes the Earth’s radius; kPLat is the latitude coordinate of the reference point; $M_{PI}$ represents the mathematical constant pi (*π*).

The longitude value of the target point was calculated as follows:

(10) $$lon=kPLon+\frac{delLon\times 180}{M_{PI}}$$

where kPLon represents the longitude coordinate of the known point.

Since the variation in latitude on the Earth’s surface is nonlinear, it was necessary to calculate the projected value corresponding to the latitude coordinate of the reference point:

(11) $$y_{t0}=R_{e}\times \log(\tan(\frac{M_{PI}}{4}+\frac{kPLat\times M_{PI}}{360}))$$

Subsequently, the projected value corresponding to the latitude coordinate of the target point was calculated as follows:

(12) $$y_{t}=y_{t0}+delY\times gRes$$

Finally, the latitude value of the target point was obtained as follows:

(13) $$lat=2\times \tan^{-1}(\exp(\frac{y_{t}}{R_{e}}))\times \frac{180}{\pi }-90$$

Therefore, navigation paths for unmanned agricultural vehicles were generated by converting the anchor pixel coordinates into latitude and longitude geographic coordinates, which contained the forward path, working path and return path.

## 3. Results and Discussion

**Figure 6:**
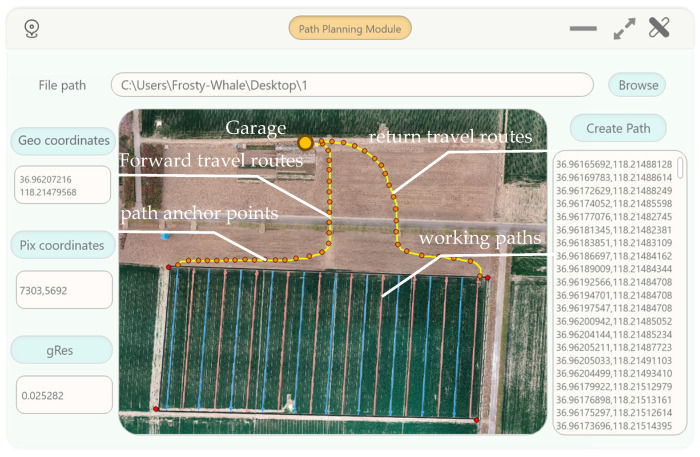
The GUI for operation.

A GUI was developed using Visual Studio 2022 to implement the UAV-based coverage path planning method in the navigation for unmanned agricultural vehicles, as shown in Figure 6. The GUI featured the function of extraction of geographic coordinate points, the design of a conversion algorithm between the pixel coordinate system and the geographic coordinate system, the automatic generation of working paths, and the creation of path anchor points along with the visualization of travel paths. The GUI generated the navigation paths according to the orthophoto map of the field obtained by the UAV.

To evaluate the accuracy and stability of the UAV-based path planning method proposed in this research, calculation accuracy tests of the path planning and field tests of the high-clearance sprayer’s navigation were conducted at Shandong University of Technology, Zibo, China.

### 3.1. Accuracy Tests of the Path Planning

**Figure 7:**
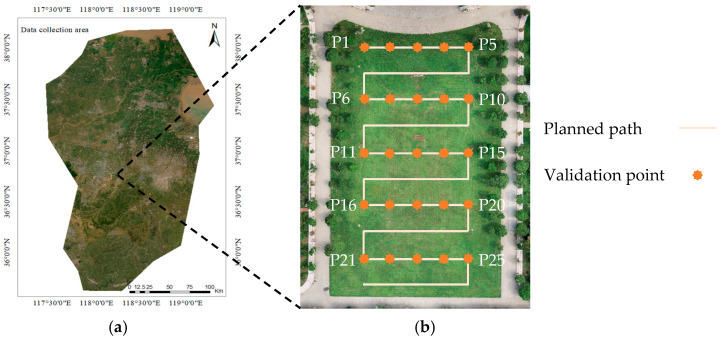
Location of the target field in (**a**) the satellite imagery and (**b**) its orthophoto map.

The flight altitude of the UAV was set at 60 m, with both head and side overlap ratios configured at 70%. A total of 136 aerial images were captured, and image stitching was performed to generate the orthophoto map of the target field, as illustrated in Figure 7. The orthophoto map of the target field was loaded into the GUI. Planned paths by the GUI using the UAV-path planning were shown in Figure 7b. Twenty-five validation points with were selected evenly on the planned path from the GUI. The geographic coordinates of these validation points were obtained through field measurements using the Bei Dou high-precision positioning system and used as reference values. The corresponding coordinates of those 25 validation points were calculated using the proposed transformation algorithm. The accuracy of the UAV-path planning was evaluated according to the position errors between the geographic coordinates and transformed coordinates of validation points.

**Figure 8:**
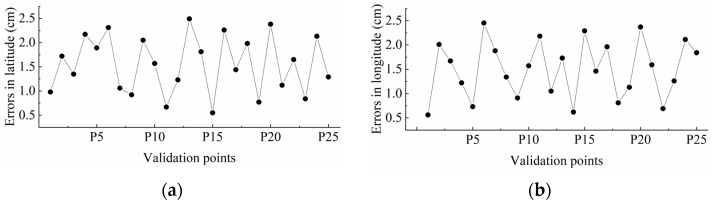
Position errors in (**a**) latitude and (**b**) longitude between two groups of validation points.

The absolute value of position errors between two coordinate groups were analyzed to quantify the transformation accuracy, as illustrated in Figure 8. Latitude errors between the reference group and test group had a maximum value of 2.49 cm with a mean value of 1.55 cm, while longitudinal errors recorded a maximum of 2.45 cm with a mean of 1.5 cm. The planar accuracy demonstrated a maximum error of 3.37 cm and a mean value of 2.23 cm. These errors were attributable to the combined effects of the accuracy of the positioning system and the limitations of the coordinate transformation. Those results indicated that the proposed coordinate transformation method has high accuracy, which met the requirement for navigation of agricultural vehicles.

### 3.2. Field Tests of the High-Clearance Sprayer’s Navigation

**Figure 9:**
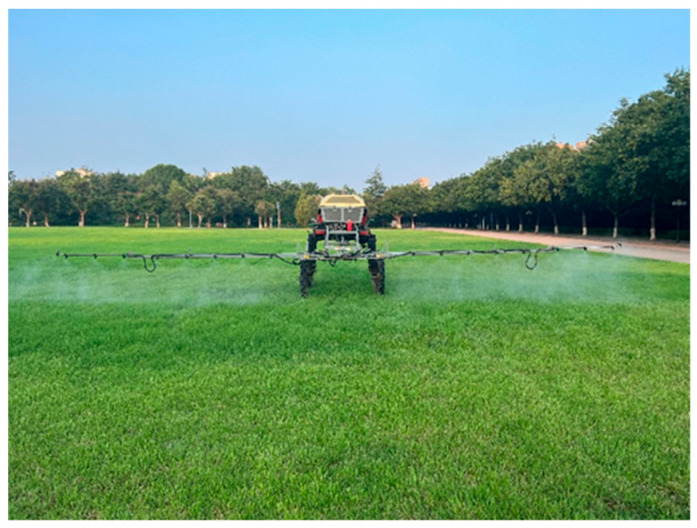
Field tests with the high-clearance sprayer of Zibo, China.

To evaluate the stability and working performance of the UAV-based path planning method proposed in this research, field tests with an unmanned high-clearance sprayer were conducted, as shown in Figure 9.

**Figure 10:**
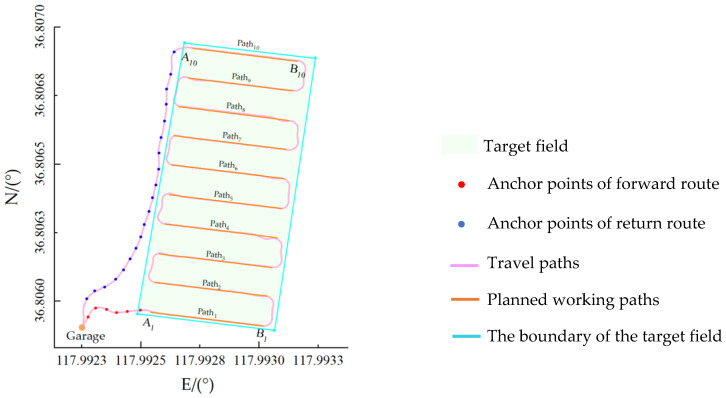
Planned path and actual navigation trajectory of the unmanned high-clearance sprayer.

A section of the target field was designated as the work place, and a position was selected as the hypothetical garage. The orthophoto map of the target field was loaded in the GUI and planned the forward path for the high-clearance sprayer from the garage to the starting point of the work place. The boundary of the work field was delineated by selecting the boundary points. Working paths for the sprayer were then designed by using the GUI’s path planning function. The return path from the ending point of the work place back to the garage was planned finally to generate the complete navigation path for the high-clearance sprayer. The automatically generated rectangular working paths consisted of 10 swaths. The navigation system of the sprayer would record the lateral errors and heading errors in real-time during field operations. Figure 10 shows the high-clearance sprayer during field operation, and Figure 10 illustrates the sprayer’s travel path and working paths.

**Figure 11:**
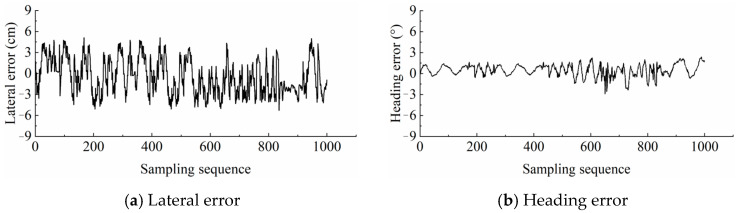
Error variations for Path 2 during field tests: (**a**) lateral error and (**b**) heading error.

In straight-line path tracking, the errors displayed a sawtooth-like pattern due to field surface undulations and steering adjustments, as shown in Figure 11. The lateral error fluctuated around 0, ranging from −5.5 cm to +5.5 cm, and the heading error fluctuated around 0 with a range from −2.5° to +2.5°.

The mean value of absolute tracking errors in a straight line was statistically analyzed using the average value, maximum value, and RMS (Root Mean Square) error as metrics to evaluate the accuracy and stability of straight-line path tracking. The statistical results of the straight-line path tracking errors are presented in Table 3.

The maximum average lateral and heading errors were 3.69 cm and 1.15°, respectively. The maximum lateral and heading errors were 5.11 cm and 1.75°, respectively. And the maximum RMS lateral and heading errors were 2.61 cm and 1.12°, respectively. For Path 1 to Path 10, the lateral error and heading error of each path were similar, with no obvious variation pattern. Field test results demonstrated that the developed UAV-based coverage path planning method was feasible and featured high accuracy, which provided an effective solution for achieving fully autonomous agricultural vehicle operations.

## 4. Conclusions

This research proposed a UAV-based coverage path planning method for unmanned agricultural vehicles to reduce manual intervention. High-resolution orthophoto maps were generated using low-altitude UAV photogrammetry. Travel paths and working paths were automatically generated according to the anchor points selected by the operator in the image coordinate domain. A Mercator projection-based coordinate transformation algorithm was designed to convert pixel-based path anchor points into geographic coordinates for generation of navigation path for agricultural vehicles. A GUI was developed to implement the proposed path planning method in the navigation of unmanned agricultural vehicles. Field tests showed that lateral navigation errors remained within ±6 cm for the unmanned high-clearance sprayer, which indicated that the developed UAV-based coverage path planning method was feasible and featured high accuracy.

Future work will focus on extending the proposed method to more complex agricultural environments, such as irregular fields and dynamic obstacles. In addition, the integration of real-time perception data and adaptive path replanning strategies will be investigated to further improve the robustness and autonomy of agricultural vehicle navigation.

## Figures and Tables

**Table 1:** Parameters of the high-clearance sprayer.

Parameters	Value
Motor power (kW)	20
Wheelbase × Thread (m × m)	1.5 × 1.5
Sprinkling width (m)	12
Traveling speed (km/h)	0–10
Tank volume (L)	500
Minimum turn radius (m)	3.5

**Table 2:** Photogrammetric remote sensing parameters of Phantom 4 RTK UAV.

Technical Parameters	Value
Camera gimbal pitch range (°)	−90–30
Camera focal length (mm)	8.8
Image resolution	4864 × 3648 (4:3)
Image sensor	1 inch CMOS

**Table 3:** Statistics of straight-line path tracking errors.

Path	Lateral Error (cm)			Heading Error (°)		
	Average	Maximum	RMS	Average	Maximum	RMS
Path 1	2.74	4.53	2.31	0.39	1.33	0.65
Path 2	2.71	4.77	2. 09	1.12	1.59	1.06
Path 3	3.69	4.25	2.18	0.72	1.67	1.11
Path 4	2.19	4.83	2.28	0.66	1.75	0.82
Path 5	2.12	4.72	2.37	1.13	1.69	0.68
Path 6	2.31	5.11	2.15	1.10	1.25	0.69
Path 7	2.28	4.32	2.61	0.52	1.41	1.05
Path 8	2.91	4.95	1.92	1.08	1.52	0.92
Path 9	2.79	4.31	1.38	0.91	1.61	0.85
Path 10	1.58	4.29	1.86	1.15	1.24	1.12

## Data Availability

Data are contained within the article.
